# The role of glycosylation in the N-terminus of the hemagglutinin of a unique H4N2 with a natural polybasic cleavage site in virus fitness *in vitro* and *in vivo*

**DOI:** 10.1080/21505594.2021.1881344

**Published:** 2021-02-04

**Authors:** Marcel Gischke, Ola Bagato, Angele Breithaupt, David Scheibner, Claudia Blaurock, Melina Vallbracht, Axel Karger, Beate Crossley, Jutta Veits, Eva Böttcher-Friebertshäuser, Thomas C. Mettenleiter, Elsayed M. Abdelwhab

**Affiliations:** a Institute of Molecular Virology and Cell Biology, Friedrich-Loeffler-Institut, Federal Research Institute for Animal Health, Greifswald-Insel Riems, Germany; b Center of Scientific Excellence for Influenza Viruses, National Research Centre (NRC), Dokki, Giza, Egypt; c Department of Experimental Animal Facilities and Biorisk Management, Friedrich-Loeffler-Institut, Federal Research Institute for Animal Health, Greifswald-Insel Riems, Germany; d California Animal Health and Food Safety Laboratory, School of Veterinary Medicine, University of California, Davis, United States; e Institute of Virology, Philipps University Marburg, Marburg, Germany

**Keywords:** Highly pathogenic, low pathogenic, avian influenza virus, h4n2, non-H5/H7, glycosylation, hemagglutinin, proteolytic activation, virulence, transmission

## Abstract

To date, only low pathogenic (LP) H5 and H7 avian influenza viruses (AIV) have been observed to naturally shift to a highly pathogenic (HP) phenotype after mutation of the monobasic hemagglutinin (HA) cleavage site (HACS) to polybasic motifs. The LPAIV monobasic HACS is activated by tissue-restricted trypsin-like enzymes, while the HPAIV polybasic HACS is activated by ubiquitous furin-like enzymes. However, glycosylation near the HACS can affect proteolytic activation and reduced virulence of some HPAIV in chickens. In 2012, a unique H4N2 virus with a polybasic HACS was isolated from quails but was LP in chickens. Whether glycosylation sites (GS) near the HACS hinder the evolution of HPAIV H4N2 remains unclear. Here, we analyzed the prevalence of potential GS in the N-terminus of HA1, ^2^NYT^4^ and ^18^NGT^20^, in all AIV sequences and studied their impact on H4N2 virus fitness. Although the two motifs are conserved, some non-H5/H7 subtypes lack one or both GS. Both sites were glycosylated in this H4N2 virus. Deglycosylation increased trypsin-independent replication in cell culture, cell-to-cell spread and syncytium formation at low-acidic pH, but negatively affected the thermostability and receptor-binding affinity. Alteration of ^2^NYT^4^ with or without ^18^NGT^20^ enabled systemic spread of the virus to different organs including the brain of chicken embryos. However, all intranasally inoculated chickens did not show clinical signs. Together, although the conserved GS near the HACS are important for HA stability and receptor binding, deglycosylation increased the H4N2 HA-activation, replication and tissue tropism suggesting a potential role for virus adaptation in poultry.

## Introduction

Avian influenza viruses (AIV) belong to the genus Influenza A Virus in the family *Orthomyxoviridae*. The genome of AIV consists of eight segments of single-stranded RNA of negative polarity [1,2] which encode more than 10 viral proteins [1]. AIV are classified according to the antigenic variants of the two surface glycoproteins, hemagglutinin (HA) and neuraminidase (NA), into 16 HA (H1 – H16) and 9 NA (N1 – N9) subtypes with 144 possible HxNy combinations. All HA and NA subtypes were isolated from wild birds, the natural reservoir of the virus [3]. Low pathogenic (LP) AIV of subtypes H5 and H7 can spontaneously mutate into highly pathogenic (HP) viruses. While most AIV subtypes are low pathogenic in the natural reservoir, transition to HP viruses mostly occurs during circulation in domestic poultry [4,5].

Although the virulence of H5 and H7 viruses is polygenically determined, mutations in the HA cleavage site (HACS) are the main determinant of virulence in poultry. Mutation of the monobasic HACS in LPAIV to a polybasic HACS in HPAIV is a prerequisite for high virulence and replication. The HA of LPAIV is activated by trypsin or trypsin-like enzymes (e.g. the human airway trypsin-like protease HAT or the transmembrane protease serine 2 TMPRSS2) in the gastrointestinal or respiratory tracts of birds. Thus, these viruses cause localized infections and mild, if any, clinical signs. Conversely, HPAIV are activated by furin-like enzymes ubiquitously expressed in all tissues causing systemic infection and death [6–8]. Intriguingly, some H5 viruses with polybasic HACS motifs exhibited low virulence in chickens [5,9]. N-Glycosylation, i.e. carbohydrate side chains attached to asparagine residues, of the HA of HPAIV plays diverse roles in protein stability, receptor binding affinity, fusion activity, immune-evasion, virulence and bird-to-bird transmission [10–15]. The N-terminal glycosylated residues near the HACS are highly conserved among all AIV subtypes [16]. The carbohydrate side chain linked to the asparagine (N) can sterically hinder the access of proteases to the HACS loop-like structure and, thus, modulate virulence by limiting access of furin-like proteases to the HACS [10,17]. A well-known natural example is the A/chicken/Pennsylvania/1983(H5N2) virus, which possessed a polybasic HACS but was avirulent in chickens. Removal of glycosylation by a mutation of asparagine in position 11 in the HA1 or increasing the number of basic amino acids (aa) in the HACS enabled trypsin-independent HA activation and a shift to high pathogenicity [10]. Conversely, removal of the same glycosylation site (GS) in a HPAIV A/Mallard/Huadong/S/2005(H5N1) revealed a significantly delayed HA cleavability and a decreased virus fitness *in vivo* and *in vitro*, while the experimentally modified GS reverted after few virus passages [18].

Some non-H5/H7 viruses exhibited high virulence after acquisition of a polybasic CS with or without reassortment with gene segments from HPAIV H5N1 [19–21]. However, it is unknown why HPAIV are restricted in nature to AIV of H5 and H7 subtypes. In each HPAIV H5/H7, acquisition of a specific polybasic HACS accompanied the shift to high virulence [5]. In 2012, an H4N2 virus was isolated from farmed quails in California with a history of 1.6% mortality [22]. Sequence analysis revealed that the HA possessed a polybasic HACS motif ^322^PEKRRTR/G^329^ [22]. This is the only known non-H5/H7 virus with 4 basic aa in the HACS, which fulfills the criteria for the HPAIV furin-specific motif (R-X-K/R-R) [23]. Nevertheless, the virus exhibited a LP phenotype in chickens without causing morbidity or mortality, and did not grow on MDCK cells without trypsin [22]. The HA possesses five potential N-linked glycosylation sites (pGS) ^2^NYT^4, 18^NGT^20, 129^NGT^131, 162^NLT^164^ and ^294^NVS^296^ in the HA1 subunit and ^482^NGT^484^ in the HA2 subunit [22], where ^2^NYT^4^ and ^18^NGT^20^ are located in the HA1 N-terminus close to the HACS region. It is unknown whether glycosylation near the HACS can affect the virulence of this unique H4N2 virus in chickens.

In this study, we analyzed pGS at positions 2 and 18 in all HA sequences of the HxNy AIV available in the Global Initiative on Sharing Avian Influenza Data (GISAID) and GenBank to 27 January 2020. Using reverse genetics, the wild-type virus (designated rgH4N2) and three recombinant H4N2 viruses with substitutions in position 2 (designated rgN2S), at position 18 (rgN18D) or at both sites (rgN2S/N18D) were rescued. The impact of the N2S and/or N18D mutations on virus fitness *in vitro* and in chickens *in vivo* was studied.

## Materials and methods

### Ethics statement

The animal experiment in this study was carried out after approval by the authorized ethics committee of the State Office of Agriculture, Food Safety and Fishery in Mecklenburg – Western Pomerania (LALLF M-V permission number 7221.3–1.1-051-12) and according to the German Regulations for Animal Welfare. The commissioner for animal welfare at the FLI representing the Institutional Animal Care and Use Committee (IACUC) approved the experiment, which was conducted in the BSL3 laboratory and animal facilities of Friedrich-Loeffler-Institut (FLI), Insel Riems, Germany.

### Sequence analysis

Sequences of the HA of AIV were retrieved from GISAID and the Influenza Virus Database of the National Centre for Biotechnology Information (NCBI). After removal of identical and laboratory-viruses, sequences were aligned using Multiple Alignment using Fast Fourier Transform (MAFFT) [24]. pGS was predicted by the sequon motif N-X-S/T, where X can be any amino acid (aa) except proline. The location of indicated aa on the tertiary structure of the HA was imposed using deduced aa sequence of HA of H4N2 virus using SWISS MODEL (http://swissmodel.expasy.org/) and then viewed in Geneious Prime (Biomatters, Version 2019.2.3) and edited manually. Numbering of aa in this study is based on the mature H4 protein after removal of the signal peptide.

### Viruses, plasmids and cells

A/quail/California/D113023808/2012(H4N2) was obtained from California Animal Health and Food Safety Laboratory System, Department of Medicine and Epidemiology, University of California, Davis. A/Victoria/1975(H3N2) was kindly provided by S. Pleschka and A. Mostafa from Justus-Liebig-University, Gießen, Germany. A/swan/Germany/R65/2006(H5N1) was obtained from the repository of the FLI kindly provided by Timm C. Harder. pHWS-plasmids containing all gene segments from this H4N2 virus were successfully cloned in a previous study [25]. pCAGGS plasmids encoding HAT and TMPRSS2 have been described previously [26]. pCAGGS plasmid was kindly provided by Stefan Finke, FLI. Plasmid expressing the GFP (pEGFP-N1, Clontech) under control of the human cytomegalovirus immediate-early 1 promoter/enhancer was kindly provided by Barbara Klupp, FLI. MDCK-HAT and MDCK-TMPRSS2 cells, which express HAT and TMPRSS2, respectively were obtained from Marburg University, and other cell lines used in this study were obtained from the Cell Culture Collection in Veterinary Medicine of the FLI. Primary chicken embryo kidney (CEK) cells were prepared from 18-day-old embryonated chicken eggs (ECE) according to the standard procedures [27].

### Generation of recombinant viruses and mutagenesis

The pHWS-HA plasmid of A/quail/California/D113023808/2012(H4N2) was used for mutagenesis using QuikChange II Site-Directed Mutagenesis Kit (Agilent, Catalog #200,523). Changing ^2^NYT^4^ and ^18^NGT^20^ to ^2^SYT^4^ and ^18^DGT^20^ to generate N2S and N18D as found naturally in some AIV, respectively, was successfully done using the following primers: N2S_F: 5´-GGGATACTCTCAGAGCTACACAGGAAATCCTGTG-´3 and N2S_R: 5´-CACAGGATTTCCTGTGTAGCTCTGAGAGTATCCC-´3 for deglycosylation at position 2 and N18S_F: 5´ CGTCATGCCGTATCTGATGGAACAATGGTAAAAACC-´3 and N18S_R: 5´-GGTTTTTACCATTGTTCCATCAGATACGGCATGACG-´3 for deglycosylation at position 18. Three HA plasmids carrying N2S, N18D or both N2S and N18D were successfully generated. Furthermore, the HA gene and mutated variants of H4N2 as indicated below were cloned from pHWS-HA into pCAGGS expression vector by conventional restriction digest and ligation. Four recombinant viruses carrying the HA from wild-type H4N2 (designated rgH4N2), N2S (designated rgN2S), N18D (designated rgN18D) or both mutations (rgN2S/N18D) and the other gene segments from rgH4N2 were rescued by transfection of MDCKII/HEK293T co-culture as previously described [28]. Two days post transfection supernatant was inoculated into 9-to-11 day-old specific pathogen free (SPF) embryonated chicken eggs (ECE) (VALO BioMedia GmbH) as recommended [29]. Allantoic fluid (AF) with a titer >16 (4log_2_) was tested for bacterial contamination on Columbia sheep blood agar. Virus stocks were stored at −70°C until use. To exclude unwanted mutations, viral RNA was extracted from virus stocks using the QIAamp Viral RNA Mini Kit and transcribed into cDNA using the Omniscript RT Kit (Qiagen, Catalog #205,111) and segments were amplified by Phusion RT-PCR kit [28]. Gel slices were extracted using Qiagen Gel Extraction kit (Qiagen, Catalog #28,706) and subjected to Sanger sequencing using ABI BigDye Terminator v.1.1 Cycle Sequencing Kit (Applied Biosystems, Catalog #4,337,452). Plaque forming units (pfu) of virus stocks were determined as described below.

### Multicycle replication kinetics

Growth kinetics were performed in CEK in 12-well plates at a multiplicity of infection (MOI) of 0.001 pfu for 8 and 24 h post-infection (hpi). Infection experiments of MDCK, MDCK-HAT and MDCK-TMPRSS2 cells were performed for 24 h only. Briefly, cells were infected for 1 h at 37°C and 5% CO2, incubated for 2 min with citric acid buffer (pH 3.0), then washed twice with 1x phosphate-buffered saline (PBS) and covered with Minimum Essential Medium (MEM) containing 0.2% bovine serum albumin (BSA) (MP Biomedicals, Catalog #9048-46-8) with antibiotics (penicillin-streptomycin) in the presence or absence of 2 µg/µl of N-tosyl-L-phenyalanine chloromethyl ketone (TPCK)-treated trypsin (Sigma Aldrich, Catalog #4,370,285). At the indicated time points, cells and supernatants were collected and stored at −70°C until used for virus titration by plaque assay as described below. All experiments were conducted in three independent replicates. Results are expressed as average and standard deviation for all replicates.

### Virus titration

Virus titration was performed by plaque assay. Briefly, confluent MDCKII cells in 12-well plates were incubated with 10-fold dilutions of recombinant viruses at 37°C and 5% CO_2_. After an hour, cells were washed twice with 1 x PBS and covered by semi-solid Bacto^TM^ Agar (BD, Catalog #90,000-767) with 50% MEM containing 4% BSA and 1 µg/µl trypsin without antibiotics. The plates were incubated for 3 days at 37°C and 5% CO_2_, and then fixed for 48 hours using 10% formaldehyde containing 0.1% crystal violet. After removal of the Bacto^TM^ Agar, plates were rinsed with distilled water, left to dry and the plaques were counted. Viral titers were expressed as plaque-forming units per ml (pfu/ml).

### Cell-to-cell spread

To study the impact of deglycosylation on cell-to-cell spread with and without trypsin, MDCKII cells were infected with recombinant viruses for 1 h and further processed as described above using plaque assay. After fixation with 10% formaldehyde containing 0.1% crystal violet, the diameter of 50 plaques was measured by microscopy using Eclipse Ti-S with software NIS-Elements, version 4.0; Nikon. Diameter of plaques of the rgH4N2 in the absence of trypsin was adjusted to 100%. The plaque size obtained by different recombinant viruses was calculated relative to the rgH4N2. Results are expressed as relative mean and standard deviation.

### Western blot

Glycosylation of the HA was confirmed using N-Glycosidase F (PNGase F, New England BioLabs, Catalog #P0704S) in standard Western Blot procedures with few modifications [30]. Briefly, cells in 12-well plates were infected at MOI of 1 for one hour. Cells were washed with PBS and MEM containing BSA with trypsin was added to each well. Infected MDCK cells were incubated for 8 h at 37°C and then collected in 2 ml Eppendorf tubes. Cells were washed twice with 1x PBS followed by centrifugation at 14,000 rpm for 15 min. Enzymatic deglycosylation by N-Glycosidase F (PNGase F, New England BioLabs, Catalog #P0704S) of N2 and N18 was done according to the producer’s guidelines. Proteins were denatured in Laemmli buffer (Serva, Catalog #42,556.01) containing 5% 2-mercaptoethanol for 5 min at 99°C. Cell lysates along with BenchMark™ Pre-Stained Protein Ladder marker were separated by discontinuous sodium dodecyl sulfate and 10% polyacrylamide gel electrophoresis (SDS-PAGE). Proteins were transferred to nitrocellulose membranes using a blotting device at 20 V for 90 min and membranes were blocked for 1 h in 5% skim milk solution. The HA protein was detected with AIV polyclonal anti-HA H4N2 antibodies derived from rabbits immunized with an H4N2 HA2 specific-peptide. The blots were incubated with primary antibodies overnight followed by washing with TBS buffer with 0.25% Tween®20. Secondary peroxidase-conjugated anti-rabbit IgG for HA (Jackson Immuno Research, Catalog #111-035-144) were used at a dilution 1:20,000. The immunodetection was done by chemiluminescence using Clarity^TM^ Western ECL Substrate (BioRad, Catalog #1,705,061). Images were acquired by a Bio-Rad Versadoc 4000 Molecular Imager (BioRad, Catalog #170-8640) and analyzed by Quantity One software (BioRad).

### Receptor-binding assay

Affinity to the avian α2,3-linked sialic acid (SA) receptors was determined by a solid-phase binding assay [31,32]. Briefly, viruses were adjusted to 10^5^ pfu/ml or 32 HA units. Plates pre-coated with 10 µg/ml fetuin (Sigma Aldrich, Catalog #F3004) were incubated with 50 μl viruses overnight at 4°C. Unbound virus was aspirated and the plates were washed with 2x PBS and blocked overnight at 4°C by 0.1% neuraminidase inactivated BSA. Afterward the plates were washed with 2x PBS + Tween® 80 (0.01%) and incubated with a 1:2 serial dilution of HRP-fetuin containing α2,3-SA for 1 h at 4°C. The plates were washed again and incubated for 30 min at room temperature (rt) in the dark. The reaction was stopped with 50 μL of 3% H_2_SO_4_ and the optical density (OD) was measured at 450 nm using Tecan ELISA Reader.

### Fusion assay

The effect of glycosylation at positions 2 and 18 on pH-dependent fusion activation of the recombinant viruses was studied as previously described [33,34], with few modifications. Briefly, QM-9 cells in 24-well plates were transfected using Lipofectamine2000® with 0.6 µg pCAGGS-plasmid containing HA from rgH4N2, N2S, N18D and N2S/N18D and GFP plasmid (100 ng/µl) to facilitate the evaluation of the assay. Transfected cells were covered with MEM containing 5% FCS, and incubated for 16 hours at 37°C and 5% CO_2_. Thereafter, supernatant was removed and the cells were treated with MEM containing BSA and 0.05% trypsin for 10 min at rt for proteolytic activation of HA. Furthermore, the cells were incubated for 15 min with MEM containing 10% FCS. Medium was removed and PBS fusion buffers previously adjusted to pH 4.0, 4.2, 4.4, 4.6, 4.8, 5.0, 5.2, 5.4, 5.6, 5.8 and 6.0 were added for 4 min. The cells were then washed with 1x PBS, and MEM with 10% FCS was added. After 4 hours incubation at 37°C, 4% paraformaldehyde (PFA) was added for 10 min and cells were washed with PBS. Syncytia formation was measured by fluorescence microscopy (Eclipse Ti-S with software NIS-Elements, version 4.0; Nikon) and the average area of syncytia was calculated. The pH threshold was the highest pH value at which fusion was observed.

### Heat stability

Viruses were adjusted to 10^5^ pfu/ml or 32 HA units and incubated at 50°C for 0, 0.5, 1, 2, 4, and 6 hours in duplicates and then stored at −70°C for titration. HA test and plaque assay were used to determine the HA activity and infectivity, respectively, as previously described [29]. The HA test and plaque assay were conducted in duplicates for each virus. The results are shown as relative average and standard deviation of all experiments for each virus.

### Histopathology and immunohistochemistry

Four 14-day-old SPF ECE were inoculated with 10^4^ pfu of each virus via the allantoic sac. Eggs were candled daily for 4 days and subsequently collected in 10% neutral buffered formalin. Tissue samples were paraffin-embedded and 2-3-μm-thick sections were stained with hematoxylin and eosin (HE). The severity of necrotizing inflammation was scored on an ordinal 0 to 3 scale: 0 = no change; 1 = mild; 2 = moderate, and 3 = severe necrosis. The distribution of recombinant viruses in different organs and tissues were studied by immunohistochemistry as described [35] using a primary antibody against the M1 protein of IAV (ATCC clone HB-64). The extent of viral antigen labeling was scored on a 0 to 3 scoring scale: 0 = no antigen, 1 = focal to oligofocal, 2 = multifocal, 3 = coalescing/diffuse.

### Chicken experiment

SPF ECE from white leghorn chickens were purchased from VALO BioMedia GmbH. Eggs were incubated at the experimental animal facilities of the FLI until hatch. Six-week-old male and female chickens were randomly allocated into four groups. Five birds were inoculated via the oculonasal (ON) route with 10^5^ pfu of each virus on both sides (~100 µL in each side). Clinical signs were monitored daily and mortality for 10 dpi. Oropharyngeal (OP) and cloacal (CL) swabs were collected at 2, 4, 7 and 10 dpi using MEM. Viral RNA was extracted from swab media using Nucleo Mag® VET Kit (Macherey & Nagel, Catalog #744,200.4) according to the manufacturer's instructions using the KingFisher Flex Purification System (Thermo Fisher Scientific, Catalog #5,400,630). The relative amount of viral load in the swabs was determined by generic real-time-reverse-transcription polymerase chain reaction (RT-qPCR) targeting the AIV Matrix gene [36]. Standard curves were generated by serial dilutions of H4N2 virus in each RT-qPCR plate. Quantification was performed by plotting the CT-value of a given sample against the known viral dilution in the standard curves. Results are expressed as equivalent log10 pfu/ml as arithmetic mean with standard deviation. At the end of the experiment, all surviving birds were euthanized after Isoflurane® (CP-Pharma, Catalog #1214) inhalation. Blood was collected and sera were tested for anti-AIV NP antibodies using ID screen Influenza Antibody Competition Multispecies kit (IDvet, Catalog #FLUACA-2P) following the instructions of the manufacturer.

## Statistics

Statistical analyses were done by GraphPad Prism 8 software (CA, Version 8.4.3). Differences in replication kinetics, receptor-binding activity, size of syncytia and heat stability experiments were analyzed using ordinary one-way ANOVA with post hoc Tukey tests. Plaque size and RT-qPCR results were evaluated using ordinary one-way ANOVA with Bonferroni correction. A p-value < 0.05 was considered significant.

## Results

### pGS at position 2 and to a lesser extent position 18 is conserved in AIV subtypes, but some non-H5/H7 viruses lack pGS at both sites

**Figure 1.**
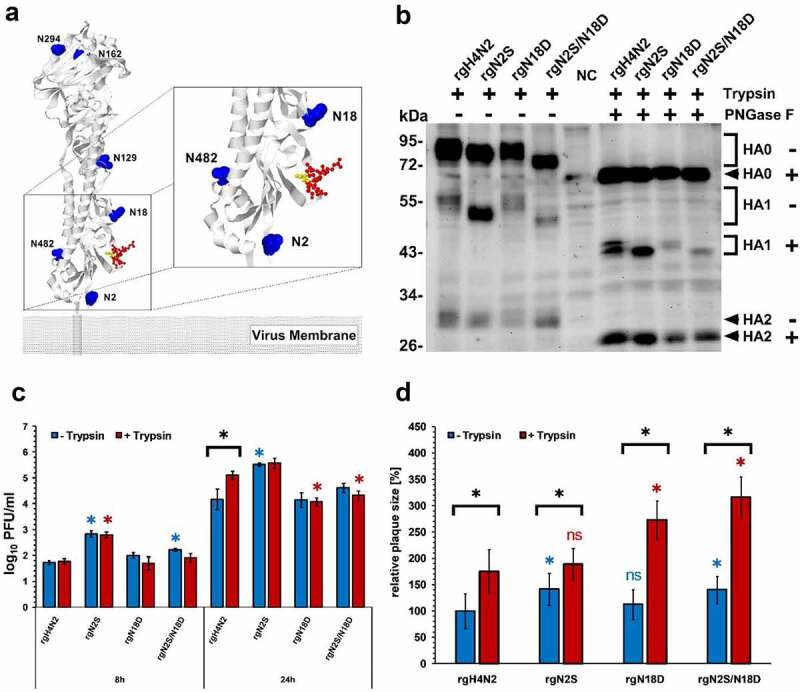
Structural modeling, expression, replication kinetics and cell-to-cell spread. Potential glycosylation sites are shown in blue, the CS is shown in red and threonine (T**R**/G) in the CS is in yellow. The model was generated by SWISS MODEL using the HA protein of H4N2 and further edited by Geneious. ^2^NYT^4^ was not found in the predicted PDB-3D structure, which starts from position 5 (a). Western Blot of HA after infection of MDCK cells with (+) or without (-) treatment with PNGase F in the presence of exogenous trypsin “T”. NC refers to negative control; naïve cells without infection (b). Replication kinetics in CEK cells at indicated time points after infection in the presence (T+) or absence (T-) of trypsin. Titration was done in MDCKII cells and the results are shown as mean ± standard deviation Log10 PFU/ml. Asterisks indicate significant differences at P < 0.05 of rgH4N2 compared to deglycosylated variants with or without exogenous trypsin (c). Cell-to-cell spread (d) was assessed by measuring 50 plaques in MDCKII with or without the addition of exogenous trypsin. Results expressed as mean and standard deviation relative to plaque size of rgH4N2 in the absence of trypsin. Asterisks indicate significant differences at P < 0.05 of rgH4N2 compared to deglycosylated variants with (red asterisk) or without trypsin (blue asterisk) and among each virus with or without the exogenous protease (black asterisk). ns = not significant compared to rgH4N2 with or without trypsin (D). Asterisks indicate significant differences at P < 0.05 of rgH4N2 compared to deglycosylated variants

Sequence analysis indicated that pGS at positions 2 and 18 are conserved in all influenza viruses with prevalence rate of 98.7% (25,787/26,114) and 66.9% (17,460/26,114), respectively. The prevalence of mutations, which lead to the loss of glycosylation at the first pGS, ranged from 0.1 to 16.1% and were highest in subtype H14 (16.1%) followed by H9 (3.2%), while the first pGS was conserved in all representatives of subtypes H1, H2, H8, H11, H12 and H15 (Supplementary Table S1). While H4, H7, H9, H10 and H12-H15 had single pGS (e.g. **NYT**G), other subtypes have overlapping pGS motifs (e.g. **NNS**T). At the second position, no pGS motif was observed in subtypes H8, H9, H12, H13 and H16. The highest prevalence for the loss of the second pGS was observed in H10 viruses (0.8%), while it was completely conserved in H14 and H15. Although the pGS at position 2 was not represented in tertiary structure, however, it is expected to localize downstream from the HACS. pGS at position 18 is located upstream from the HACS and glycosylation there may sterically hinder the access of proteases (Figure 1 panel A). This sequence analysis indicates that the HA pGS at position 2 is highly conserved in most of AIV subtypes, whereas the second pGS (i.e. equivalent to position 18) was absent in five different subtypes. Some non-H5/H7, particularly H9N2, lack both pGS.

### Generation of four recombinant viruses

Four recombinant viruses were successfully constructed and propagated. Virus titer was determined by plaque assay to 5 × 10^5^ to 2 × 10^6^ pfu/ml.

### ^2^NYT^4^ and ^18^NGT^20^ are glycosylated

Status of glycosylation was determined by treatment of the infected MDCK-cell lysates with PNGase F, which removes all N-linked glycosylation including high mannose, hybrid, bi-, tri- and tetra-antennary variants. Without PNGase F treatment, the molecular size of HA0 and HA1 of the four viruses differed, while HA2 had a similar size among all viruses indicating that variation in the size of HA0 is due to variation in the HA1 molecular weight. The HA0 of rgN2S showed a lower molecular weight compared to rgH4N2. The size of HA0 and HA1 of rgN18D were slightly lower than rgH4N2, whereas the size of rgN2S/N18D was clearly lower than rgH4N2 and rgN2S (Figure 1 Panel B). After treatment, there was no difference in the molecular weights of HA from all viruses, indicating the removal of all GS in the HA0 and a remarkably decreased shift in HA1. The HA1 of rgN2S and rgN18D had similar molecular weight indicating that the shift seen in the HA1 of rgN18D without PNGase treatment was due to glycosylation. These results indicate that both sites are glycosylated (Figure 1 panel B).

### Mutation of GS at position 2 increased trypsin-independent replication of rgH4N2 in CEK

Replication of the recombinant viruses in the presence or absence of trypsin was studied in CEK. All viruses replicated in the presence or absence of exogenous trypsin (Figure 1 panel C). The addition of trypsin increased the replication of rgH4N2 to a significantly higher level than without trypsin at 24 hpi (p < 0.001) (Figure 1 panel C). The addition of trypsin did not significantly affect the replication titers of the other viruses (Figure 1 panel C). Compared to rgH4N2, rgN2S replicated at significantly higher levels at 8 with or without trypsin and 24 hpi without trypsin (p < 0.0001) and insertion of N2S/N18D increased virus replication at significantly higher levels at 8 hpi without trypsin (p < 0.03) (Figure 1 panel C). N18D did not affect the replication of rgH4N2 in CEK cells (Figure 1 panel C). These results indicate that N2S increased trypsin-independent replication of rgH4N2 in CEK.

### Mutation of GS at position 2 increased cell-to-cell spread with or without trypsin

To study the impact of deglycosylation on cell-to-cell spread of H4N2, plaque sizes in MDCKII cells with or without trypsin was measured (Figure 1 panel D). In the absence of trypsin, rgN2S and rgN2S/N18D produced significantly larger plaques than rgH4N2 (p < 0.0008). Trypsin significantly increased the size of plaques induced by each virus (p < 0.0001) and the largest plaques were induced by rgN18D and rgN2S/N18D (p < 0.0001). These data indicate that N2S increased cell-to-cell spread but trypsin is still required for efficient cell-to-cell spread of all viruses.

### In MDCK-HAT and MDCK-TMPRSS2 cells all viruses replicated at similar levels

**Figure 2.**
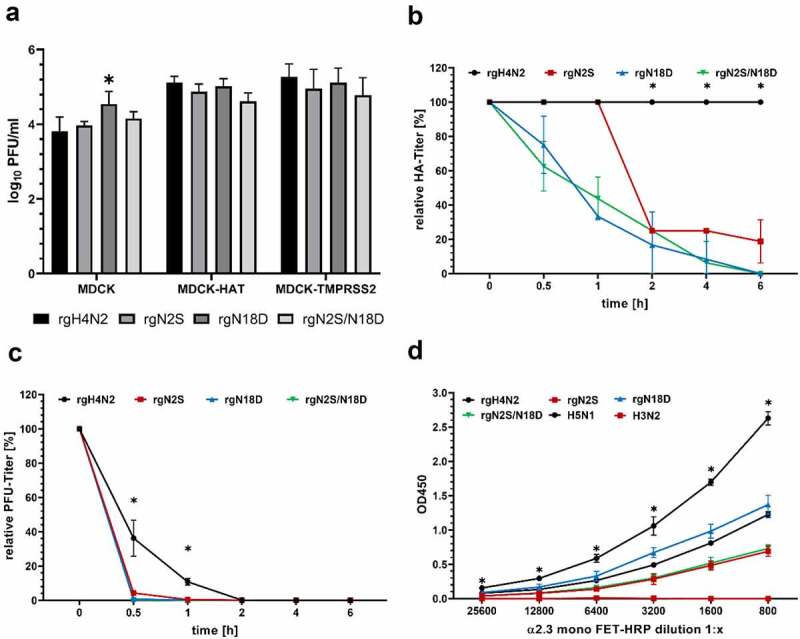
Impact of deglycosylation on replication, thermostability and receptor binding affinity. Multicycle replication in the cells and supernatant 24 hour post infection (hpi) of MDCK, MDCK-HAT and MDCK-TMPRSS2 cells. Asterisks indicate significant differences at P < 0.05 of rgH4N2 compared to deglycosylated variants (a). Heat stability at 50°C after indicated time points of all rgH4N2 variants considered in this study were tested based on HA (b) or PFU (c) titers. Asterisks indicate significant difference at P value < 0.05 of rgH4N2 compared to deglycosylated variants. Receptor binding assay after adjustment of the viruses to HA titers (d) was performed in 4 replicates to identify the affinity to the avian α2,3-linked sialic acid receptors. Avian H5N1/R65 and human H3N2 virus were used as positive and negative control, respectively

To study the possible impact of the trypsin-like enzymes HAT and TMPRSS2 on virus replication, MDCK-HAT and MDCK-TMPRSS2 cells were infected with different viruses and virus titers in collected cells and supernatant were determined by plaque assay compared to MDCK cells (which do not produce endogenous HAT or TMPRSS2) (Figure 2 panel A). In MDCK cells, all viruses replicated at similar levels, while rgN18D replicated to significantly higher titers than rgH4N2. In MDCK-HAT and MDCK-TMPRSS2 cells, all viruses replicated to comparable levels which were slightly higher than replication titers in MDCK cells, although it was not statistically significant (p > 0.057) (Figure 2 panel A). These data suggest that deglycosylation did not significantly affect virus replication in the presence of TMPRSS2 and HAT.

### Deglycosylation reduced thermostability of rgH4N2

The impact of deglycosylation on thermostability at 50°C was studied. HA of rgH4N2 was stable for 6 hours as determined by HA assay and plaque assay (Figure 2). Conversely, deglycosylation dramatically decreased the HA activity of rgN18D and rgN2S/N18D to 82 and 63% after 30 minutes, and after 1 h both viruses had only 36 and 44% activity, respectively. From 2 hours, deglycosylation significantly decreased HA activity of rgN2S compared to rgH4N2 (p < 0.0001) (Figure 2 panel B). Furthermore, all viruses showed a remarkably reduced infectivity in MDCKII cells already after 30 minutes. However, the infectivity of rgH4N2 was significantly more stable after 30 minutes (p < 0.0001) and 1 h (p < 0.0001) than observed for the deglycosylated variants (Figure 2 panel C). These results indicate that glycosylation is important for thermostability of HA H4N2 at elevated temperature.

### Deglycosylation reduced binding affinity to avian-type receptors

The affinity of different viruses to avian-type receptors was studied using a solid-phase binding assay against fetuin labeled with α2,3-SA. H4N2 showed strong binding affinity to avian-type receptors compared to other viruses. Human H3N2 did not bind effectively to avian-type receptors. Deglycosylation at positions 2 and/or 18 clearly reduced the binding affinity to avian-type receptors; although at higher or comparable levels to HPAIV H5N1 (Figure 2 panel D). These data indicate that deglycosylation reduced H4N2 affinity to avian-type receptors; however, the binding affinity was still comparable to HPAIV H5N1.

### Deglycosylation increased cell fusion at low-acidic pH

**Figure 3.**
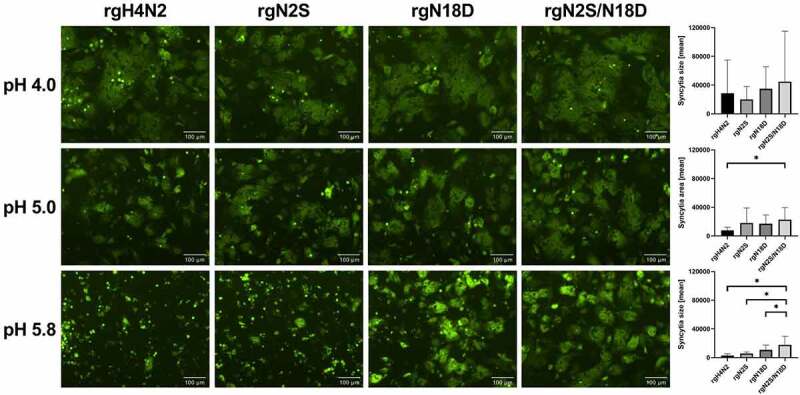
Impact of deglycosylation to trigger fusion at different pH values. Fluorescence microscopy of syncytia formation in QM-9 cells after expression of HA of rgH4N2 and HA carrying N2S, N18D or N2S/N18D as well as GFP, and activation at pH of 4.0, 5.0 and 5.8. Bars = 100 µm. Syncytia were measured and the average area and standard deviation for each virus is shown. Asterisks indicate significance difference at p < 0.05

The impact of deglycosylation on pH activation for HA-cell membrane fusion was analyzed by analysis of syncytia formation in quail fibrosarcoma (QM9, CCLV-RIE 466) cell line at different pH values between 4.0 and 6.0. While high-acidic pH (pH = 4) activation revealed no statistical difference in cell fusion among all glycosylation variants, statistical differences in syncytia formation were observed at low-acidic pH values (Figure 3). HA of rgH4N2 showed fusion activity at pH 4.0 to 5.0, while HA of rgN2S was still activated at pH values slightly higher pH 5.4. Syncytia formation for N18D and N2S/N18D was observed from pH 4.0 to 5.8. Together, deglycosylation, particularly N18D, enabled fusion-activation of the HA at low-acidic pH conditions.

### Deglycosylation expanded the tissue tropism of H4N2 in chicken embryos

**Figure 4.**
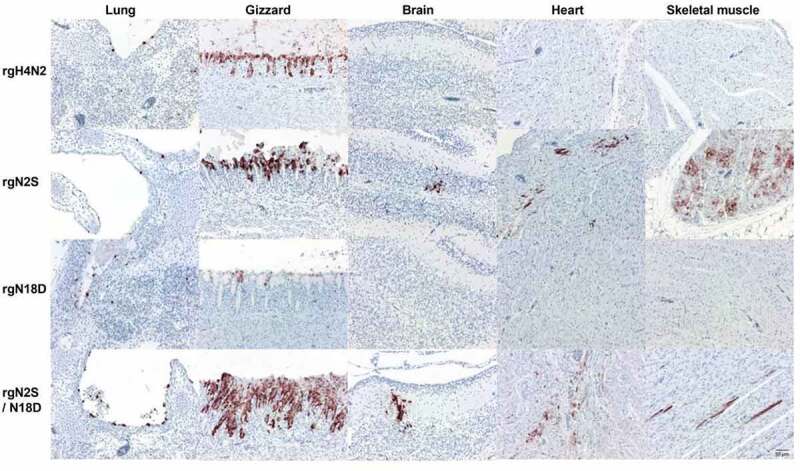
Distribution of avian influenza virus matrix protein (M1) in selected organs of chicken embryos. Distribution of influenza M1 protein in the indicated organs of embryos inoculated with different viruses. 14-day-old embryonated chicken eggs were inoculated via the allantoic sac for 5 days then embryos were subjected to immunohistochemistry using primary anti-matrix antibody for antigen detection. Labeling by 3-amino-9-ethyl-carbazol (red-brown); hematoxylin counterstain (blue). Bars = 50 µm

To determine the histopathological changes and distribution of viruses in different tissues, organs of inoculated chicken embryos were subjected to histopathological and immunohistological examination. Necrosis in the gizzard was observed in all groups (Figure 4). Inoculation with rgH4N2 led to viral antigen detection restricted to superficial epithelia in several organs, i.e. the skin, respiratory tract (beak, trachea, lung), gastrointestinal tract (proventriculus, gizzard, small and large intestine), and bursa. Most abundantly, antigen was found in the gizzard and proventriculus (coalescent to diffuse), all other tissues exhibited focal to multifocal viral antigen. The same distribution pattern and abundancy was found for rgNS2, but additionally, focal to multifocal labeling was detected in the parenchyma of the pancreas, heart, skeletal muscle, and brain. In addition to the findings for rgNS2, rgN2S/N18D was found in the tubular epithelium of the kidney and in scattered endothelial cell in several organs. In contrast, rgN18D reflected the pattern of rgH4N2 but labeling never exceeded single positive foci. Viral antigen detection was associated with mild to moderate, acute necrosis as well as deceased cellularity within the lymphoid follicles of the bursa after inoculation with rgH4N2, rgN2S, or rgN2S/N18D. rgN18D led to mild necrosis restricted to the proventriculus and gizzard. Taken together, rgN2S particularly in combination with rgN18D led to a more widespread tissue tropism, while rgN18D alone restricted virus replication and lesions.

### Deglycosylation did not alter virulence of the virus and virus excretion in chickens

**Figure 5.**
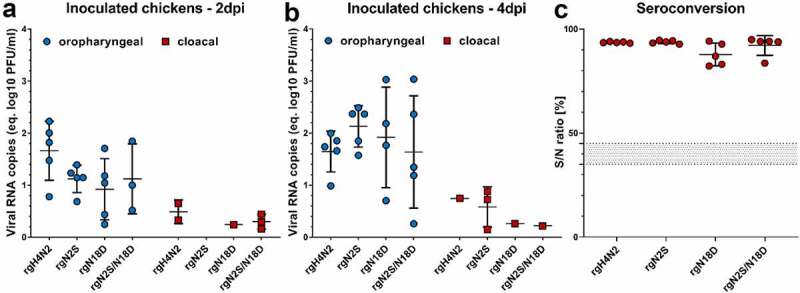
Virus excretion in oropharyngeal and cloacal swabs and seroconversion after intranasal inoculation of chickens. Virus excretion in oropharyngeal and cloacal swabs collected at 2 (a) and 4 (b) dpi in inoculated chickens was determined by RT-qPCR targeting the M gene. Results are shown as viral RNA copies (equivalent log10 PFU/ml) with average ± standard deviation. Anti-NP antibodies were determined by inhibition ELISA in serum samples collected from all chickens at the end of the experiment (c). Shown are the average S/N ratio and standard deviation of positive samples

Chickens were inoculated with each virus via the oculonasal (ON) route. All inoculated birds remained healthy without clinical signs or mortality. Virus excretion in OP and CL swabs at 2 and 4 dpi was analyzed by RT-qPCR (Figure 5 panels A and B). In OP swabs, the RNA was detected in all inoculated chickens except two rgN2S/N18D inoculated-chickens at 2 dpi, which were tested negative. Nevertheless, there was no significant difference in the amount of virus excretion in OP swabs between different groups (Figure 5 panel A). Cloacal shedding was determined in low amounts, if at all, on both days (Figure 5 panel B). In CL swabs, at 2 dpi, the viral RNA was detected in inoculated chickens with rgH4N2 (2/5), rgN18D (1/5) and rgN2S/N18D (3/5). Conversely, at 4 dpi, rgN2S was detected in 3/5 birds and only 1/5 birds in other groups were positive. Trials to re-isolate the virus or direct sequencing in swab samples in this experiment were not successful. All inoculated chickens possessed anti-NP antibodies as tested by ELISA (Figure 5 panel C). These results indicate that deglycosylation around the HACS did not significantly affect low virulence or excretion of the H4N2 with a unique natural polybasic cleavage site in chickens.

## Discussion

HPAIV H5 and H7 evolve from LP precursors after mutation of the monobasic HACS to a polybasic motif. Non-H5/H7 viruses with a polybasic CS are very rare in nature. In 2012, a unique H4N2, the only non-H5/H7 with 4 basic aa (^322^PEKRRTR/G^329^) in the HACS was isolated from quails, but the virus exhibited low virulence in chickens [22]. In contrast to H5 and H7 viruses, no data are available on the role of glycosylation in the HA1 N-terminus of non-H5/H7 viruses. Whether removal of conserved GS in the vicinity of the HACS affects virus fitness and subsequently replication and virulence in chickens was studied herein.

In this study, sequence analysis of 26,114 HA proteins obtained from GenBank and GISAID showed that pGS at positions 2 and 18 were conserved across AIV HA subtypes, particularly those adapted to poultry, except H9 viruses which lack glycosylation at position 18 which is partially similar to a previous report [37]. Remarkably, more than 240 H9 viruses lack pGS at both sites indicating that they might be dispensable for replication. We showed that asparagine at positions 2 and 18 are glycosylated which is in accordance with results on the analogous positions in some H5 and H7 viruses [12,15,18]. It is known that trypsin and trypsin-like enzymes are important for multiple cycle replication of LPAIV. However, rgH4N2 in this study replicated to a certain level in CEK cells and spread from cell-to-cell in MDCKII without trypsin. Both cell lines have endogenous proteases (e.g. matriptase) which may activate some LPAIVs [38]. Moreover, furin-like enzymes or proteases in the allantoic fluid during propagation of viruses in ECE probably supported the growth of rgH4N2 without trypsin as previously described [39]. It is worth to mention that trypsin-independent replication of LPAIV H6N1 and H7N7 in MDCK, MDCKII and/or CEK cells has been previously reported [20,40]. Deglycosylation of N2S (alone or in combination with N18D) increased virus replication in CEK cells and cell-to-cell spread in MDCKII cells without trypsin. Conversely, the removal of a similar GS ^11^NST^13^ in an HPAIV H5N1 or HPAIV H7N1 impaired activation of HA0 and decreased virus growth in cell culture [18,41]. Glycosylation at position 28 (equivalent to N18D in H4N2) was indispensable for the infectivity of HPAIV H7N1 [41].

HA0 is a fusion-inactive protein which must be cleaved by host proteases into the HA1/HA2 two subunit complex to expose the fusion peptide in the HA2 [7]. The fusion of influenza viruses with host cell membranes is pH-dependent which results in irreversible conformational changes. The fusion alters intracellular host cell responses (e.g. IFN response) and subsequently regulates infectivity. The pH value of the early endosome is about 6 to 6.3 and in the late endosome is 5 to 6 (reviewed by [42]). Thus, rapid fusion may enhance early virus replication before triggering the host-immune response. However, pH stability is also important for persistence in the environment. Therefore, AIV vary in their optimal range for pH-triggered fusion/activation from 4.4 to 6.4 [42,43]. In this study, rgH4N2 showed optimal pH-triggered activation at high acidic pH (pH ≤ 5). In the presence of trypsin, the N18D mutation alone or in combination with N2S triggered syncytia formation at low acidic pH (5.8). Similar observation for pH fusion activation has been reported after removal of GS in position 28 (equivalent to position 18 in H4N2) in the HA stem domain of A/FPV/Rostock/34 (H7N1) [15]. Hence, the GS at position 18 is located closer to the middle of the HA stem domain than the site at position 2. It seems possible that oligosaccharides at position N18 are required for stabilization of the metastable HA during virus-host membrane fusion [15]. Moreover, it is known that glycosylation is important for stability of the HA protein especially at higher temperature (e.g. intestinal tract of birds, fever or at ambient temperature) [44]. In this study, mutations N2S or N18D dramatically affected HA activity and infectivity. Similar results were observed for A/Mallard/Huadong/S/2005 H5N1 after removal of the glycosylation at position ^11^NST^13^ indicating an impact on structural stability by glycosylation at the N-terminus [18]. Interestingly, H4N2 had a stronger affinity to α2,3 SA receptors than H5N1. Deglycosylation at positions 2 and 18 significantly reduced binding affinity to α2,3 SA indicating conformational changes in the head domain. Similar results have been described after deglycosylation at ^11^NST^13^ of an H5 virus [45]. However, this reduction in receptor affinity was similar to HPAIV H5N1 and apparently sufficient for efficient virus replication in chicken cells or embryos.

Chicken embryos as a model have been previously used to assess tissue distribution and virulence of different influenza viruses [35,46–48]. In this study, the rgH4N2 viral antigen distribution was restricted to superficial epithelia mainly in the respiratory and gastrointestinal tract and was associated with necrosis. Deglycosylation N18D restricted the viral tropism and lesions whereas deglycosylation N2S or N2S/N18D led to a more systemic spread and lesions profile, also affecting the brain, heart, skeletal muscles and kidneys. These data indicate that deglycosylation N2S is advantageous for systemic virus tropism in chicken embryos. In chickens, rgH4N2 exhibited low virulence despite of the polybasic cleavage site which is similar to previous findings [22,25]. In contrast to these results, deglycosylation at position 11 (equivalent to position 2 in H4N2) reduced virulence of HPAIV H5N1 in intravenously inoculated 6-week-old chickens [18].

In conclusion, H4N2 with a unique polybasic cleavage site and mutation of GS at position 2 and 18 exhibited increased trypsin-independent replication in chicken cells, increased cell-to-cell spread, triggered fusion activation at low acidic pH and enabled systemic spread in chicken embryos, which were driven mostly by the N2S mutation. Both mutations reduced receptor binding and thermostability. No impact on virulence in chickens was observed, however. Despite the important role of glycosylation in the vicinity of H4N2 HACS for HA stability, removal of the glycosylation at position 2 may play a role in adaptation of non-H5/H7 which is in contrast to similar studies using HPAIV H5N1 or H7N1.

## Supplementary Material

Supplemental Material
