# Supplementary material for: The role of glycosylation in the N-terminus of the hemagglutinin of a unique H4N2 with a natural polybasic cleavage site in virus fitness in vitro and in vivo
Source: Virulence. 2021 Feb 4;12(1):666–78. doi: 10.1080/21505594.2021.1881344 (PMC7872060; doi:10.1080/21505594.2021.1881344)
Supplement: Supplemental Material [file KVIR_A_1881344_SM4908.docx]

**Supplementary Table S1:** Prevalence of potential N-glycosylation in the N-terminus of HA1 in different AIV subtypes

| **Subtype Hx** | **N2*** | **N18** |
| --- | --- | --- |
| H1 | **100%** (808/808) | **99.6%** (811/814) |
| H2 | **100%** (504/504) | **99.8%**(503/504) |
| H3 | **98.9%** (1832/1852) | **99.5%** (1882/1892) |
| H4 | **98.1%** (1424/1452) | **99.9%** (1461/1463) |
| H5 | **99.9%** (6881/6886) | **99.7%** (6925/6948) |
| H6 | **99.9%** (2012/2014) | **99.9%** (2019/2021) |
| H7 | **99.2%** (2220/2239) | **99.5%** (2228/2240) |
| H8 | **100%** (204/204) | **0%** (0/204) |
| H9 | **96.8%** (7415/7657) | **0%** (0/7657) |
| H10 | **99.5%** (772/776) | **99.2%** (777/783) |
| H11 | **100%** (811/811) | **99.8%** (809/811) |
| H12 | **100%** (335/335) | **0%** (0/335) |
| H13 | **99.7%** (363/364) | **0%** (0/364) |
| H14 | **83.9%** (26/31) | **100%** (31/31) |
| H15 | **100%** (14/14) | **100%** (14/14) |
| H16 | **99.4**% (166/167) | **0%** (0/167) |
| **Total** | **98.7% (**25787/26114) | **66.9%** (17460/26114) |

* Percent of number of sequences with a potential N-glycosylation site/total number of sequences retrieved from GISAID and GenBank to 27-01-2020. The prevalence of N-linked glycosylation at the first two positions in HxNx viruses (equivalent to H4 positions N2 and N18) was predicted by N-X-S/T motif, where X is any amino acid except proline.
